# *catena*-Poly[[aqua­(3-methyl­benzoato-κ^2^ *O*,*O*′)lead(II)]-μ-3-methyl­benzoato-κ^4^ *O*:*O*,*O*′:*O*′]

**DOI:** 10.1107/S1600536809019771

**Published:** 2009-06-06

**Authors:** Jun Dai, Juan Yang, Xiaobing An

**Affiliations:** a Institute of Safety Science and Engineering, Henan Polytechnic University, Jiaozuo 454003, People’s Republic of China; b Department of Physical Chemistry, Henan Polytechnic University, Jiaozuo 454003, People’s Republic of China

## Abstract

The reaction of lead(II) acetate and 3-methyl­benzoic acid (MBA) in aqueous solution yielded the title polymer, [Pb(C_8_H_7_O_2_)_2_(H_2_O)]_*n*_. The asymmetric unit contains two Pb^II^ atoms, four MBA ligands and two water mol­ecules. Each Pb^II^ cation is hepta­coordinated and chelated by four carboxyl­ate O atoms from two MBA ligands. The Pb atoms are bridged through the carboxyl­ate O atoms from another two MBA ligands, leading to a central Pb_2_O_2_ core. The Pb—O bond lengths are in the range 2.325 (3)–2.757 (4) Å. The intra- and inter­dimer Pb⋯Pb distances are 4.2942 (3) and 4.2283 (3) Å, respectively, indicating little direct metal–metal inter­action. The coordinating water mol­ecules and carboxyl­ate O atoms are involved in extensive O—H⋯O hydrogen-bonding inter­actions. The complex has an extended ladder-like chain structure and the chains are assembled by hydrogen bonds and π–π inter­actions [centroid–centroid distance = 3.6246 (3) Å] into a three-dimensional supra­molecular structure.

## Related literature

For general background to metal-organic frameworks and their applications, see: Hamilton *et al.* (2004 ▶); Meng *et al.* (2003 ▶); Fan & Zhu (2006 ▶); Wang *et al.* (2006 ▶); Masaoka *et al.* (2001 ▶). For related structures, see: Shi *et al.* (2007 ▶).
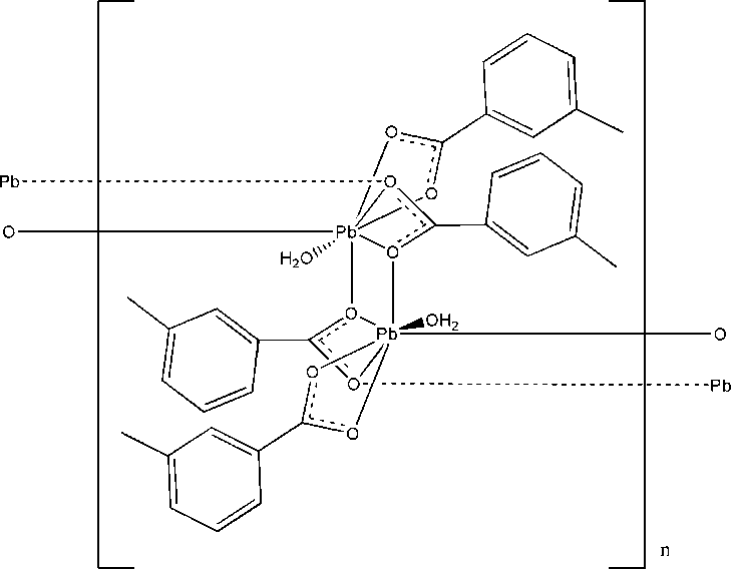


## Experimental

### 

#### Crystal data

[Pb(C_8_H_7_O_2_)_2_(H_2_O)]
*M* *_r_* = 495.48
Monoclinic, 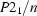

*a* = 7.1745 (3) Å
*b* = 42.745 (2) Å
*c* = 10.7126 (5) Å
β = 90.765 (1)°
*V* = 3285.0 (3) Å^3^
*Z* = 8
Mo *K*α radiation
μ = 10.29 mm^−1^
*T* = 296 K
0.36 × 0.17 × 0.12 mm

#### Data collection

Bruker APEXII CCD area-detector diffractometer
Absorption correction: multi-scan (*SADABS*; Bruker, 2007 ▶) *T*_min_ = 0.144, *T*_max_ = 0.300
40611 measured reflections
8096 independent reflections
6265 reflections with *I* > 2σ(*I*)
*R*_int_ = 0.056

#### Refinement

*R*[*F* ^2^ > 2σ(*F* ^2^)] = 0.035
*wR*(*F* ^2^) = 0.061
*S* = 1.03
8096 reflections
397 parameters
H-atom parameters constrained
Δρ_max_ = 0.87 e Å^−3^
Δρ_min_ = −1.00 e Å^−3^

### 

Data collection: *APEX2* (Bruker, 2007 ▶); cell refinement: *SAINT* (Bruker, 2007 ▶); data reduction: *SAINT*; program(s) used to solve structure: *SHELXS97* (Sheldrick, 2008 ▶); program(s) used to refine structure: *SHELXL97* (Sheldrick, 2008 ▶); molecular graphics: *SHELXTL* (Sheldrick, 2008 ▶); software used to prepare material for publication: *SHELXTL*.

## Supplementary Material

Crystal structure: contains datablocks global, I. DOI: 10.1107/S1600536809019771/fj2218sup1.cif

Structure factors: contains datablocks I. DOI: 10.1107/S1600536809019771/fj2218Isup2.hkl

Additional supplementary materials:  crystallographic information; 3D view; checkCIF report

## Figures and Tables

**Table 1:** Selected bond lengths (Å)

Pb1—O4	2.386 (3)
Pb1—O1	2.424 (3)
Pb1—O3	2.594 (3)
Pb1—O5	2.603 (3)
Pb1—O2	2.622 (4)
Pb1—O9	2.724 (4)
Pb1—O6^i^	2.751 (3)
Pb2—O6	2.325 (3)
Pb2—O8	2.494 (4)
Pb2—O3^ii^	2.538 (3)
Pb2—O7	2.565 (4)
Pb2—O10	2.665 (3)
Pb2—O4	2.712 (3)
Pb2—O5	2.757 (4)

Symmetry codes: (i) 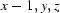; (ii) 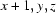.

**Table 2:** Hydrogen-bond geometry (Å, °)

*D*—H⋯*A*	*D*—H	H⋯*A*	*D*⋯*A*	*D*—H⋯*A*
O9—H9*A*⋯O8	0.82	2.03	2.805 (5)	158
O9—H9*B*⋯O7^i^	0.82	2.25	3.017 (5)	156
O10—H10*B*⋯O2	0.82	2.12	2.881 (5)	153
O10—H10*A*⋯O1^ii^	0.82	1.97	2.774 (5)	166

Symmetry codes: (i) 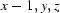; (ii) 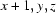.

**Fig. 1.**
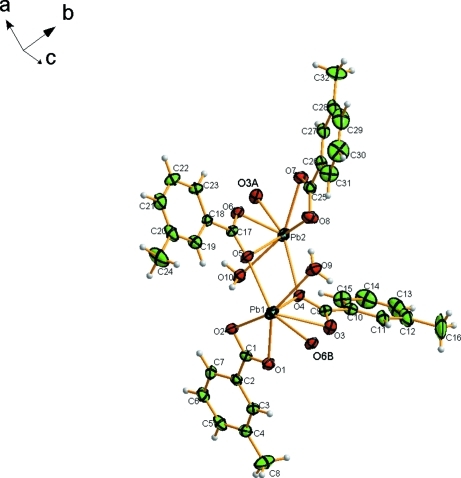
The coordination environment around Pb(II) in the title complex with the atom-labeling scheme. Displacement ellipsoids for non-hydrogen atoms are drawn at the 30% probability level.

**Fig. 2.**
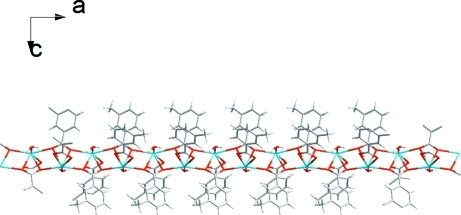
The extended ladder-like chain structure of the title compound.

**Fig. 3.**
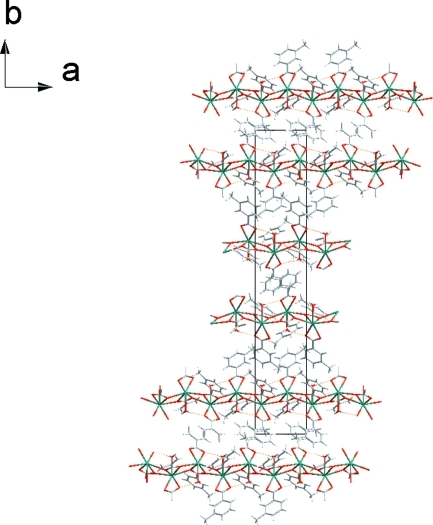
The two-dimensional layer structure of the title compound.

**Fig. 4.**
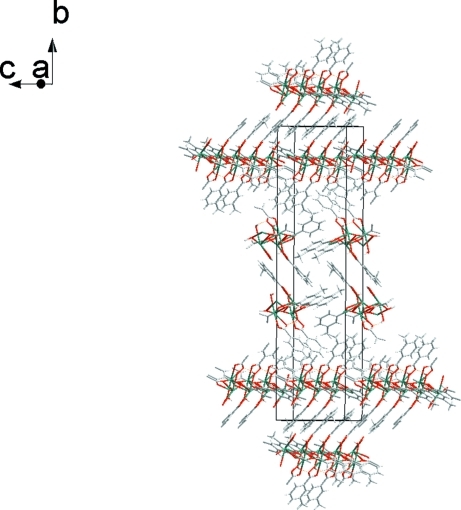
The three-dimensional structure of the title compound.

## supplementary crystallographic information

### Comment

Interest in porous metal-organic frameworks (MOFs) has been driven by the prospect of generating a wide range of materials with useful properties for applications such as ion-exchange, nonlinear optics and catalysis (Hamilton *et al.*, 2004; Meng *et al.*, 2003; Fan *et al.* 2006). On the other hand, lead(II) compounds have been increasingly studied (Shi *et al.* 2007) owing to their possible applications in different fields, especially in environmental protection due to the toxicity of lead and in biological systems for its diverse interactions with biological molecules. As an important family of multidentate O-donor ligands, aromatic carboxylate ligands have been extensively employed in the preparation of metal-organic complexes because of their potential properties and intriguing structural topologies (Wang *et al.*, 2006; Masaoka *et al.* 2001). Herein, we report the structure of the title complex.

The asymmetric unit of the title complex, [Pb_2_(C_8_H_7_O_2_)_4_(H_2_O)_2_]_n_, contains two Pb^II^ cation, four MBA ligands and two coordinating water molecule, as illustrated in Fig. 1. The two Pb atoms are connected *via* two bridging O atoms belonging to two MBA ligands, resulting the central Pb_2_O_2_ core tetratomic ring. The Pb—O bond lengths are in the range of 2.325 (3) to 2.757 (4) Å (Table 1). The average distance of two Pb atoms is 4.2942 Å, which leads to the weak metal-metal interactions. This coordination polymer structure presents extended ladder-like chain along the *a* axis direction. The coordinating water molecules and carboxylate O atoms are involved in extensive O—H···O hydrogen-bonding interactions (Table 2). These chains are assembled by H-bonds and π-π interactions to three-dimensional supramolecular structure.

### Experimental

A mixture of Pb(CH_3_COO)_2_ 3H2O (0.1992 g, 0.52 mmol), MBA (0.1139 g, 0.84 mmol), melamine (0.0255 g, 0.20 mmol) and distilled water (10 ml) was sealed in a 25 ml Teflon-lined stainless autoclave (Shi *et al.* 2007). The mixture was heated at 373 K for 5 days to give colorless  crystals suitable for X-ray diffraction analysis.

### Refinement

All H atoms bounded to C atoms were positioned geometrically and allowed to ride on their parent atoms, with C—H distances in the range of 0.93–0.96 Å. The positions of the water H atoms were found from a difference Fourier map and refined with distance restraints O—H = 0.82 Å, *U*_iso_(H) = 1.2Ueq(O).

### Crystal data

**Table:**

[Pb(C_8_H_7_O_2_)_2_(H_2_O)]	*F*(000) = 1872
*M**_r_* = 495.48	*D*_x_ = 2.004 Mg m^−^^3^
Monoclinic, *P*2_1_/*n*	Mo *K*α radiation, λ = 0.71073 Å
Hall symbol: -P 2yn	Cell parameters from 7418 reflections
*a* = 7.1745 (3) Å	θ = 2.4–25.2°
*b* = 42.745 (2) Å	µ = 10.29 mm^−^^1^
*c* = 10.7126 (5) Å	*T* = 296 K
β = 90.765 (1)°	Block, colourless
*V* = 3285.0 (3) Å^3^	0.36 × 0.17 × 0.12 mm
*Z* = 8	

### Data collection

**Table:**

Bruker APEXII CCD area-detector diffractometer	8096 independent reflections
Radiation source: fine-focus sealed tube	6265 reflections with *I* > 2σ(*I*)
graphite	*R*_int_ = 0.056
φ and ω scans	θ_max_ = 28.2°, θ_min_ = 1.9°
Absorption correction: multi-scan (*SADABS*; Bruker, 2007)	*h* = −9→9
*T*_min_ = 0.144, *T*_max_ = 0.300	*k* = −56→56
40611 measured reflections	*l* = −14→14

### Refinement

**Table:**

Refinement on *F*^2^	Secondary atom site location: difference Fourier map
Least-squares matrix: full	Hydrogen site location: inferred from neighbouring sites
*R*[*F*^2^ > 2σ(*F*^2^)] = 0.035	H-atom parameters constrained
*wR*(*F*^2^) = 0.061	*w* = 1/[σ^2^(*F*_o_^2^) + (0.0182*P*)^2^ + 3.7836*P*] where *P* = (*F*_o_^2^ + 2*F*_c_^2^)/3
*S* = 1.03	(Δ/σ)_max_ = 0.001
8096 reflections	Δρ_max_ = 0.87 e Å^−^^3^
397 parameters	Δρ_min_ = −1.00 e Å^−^^3^
0 restraints	Extinction correction: *SHELXL97* (Sheldrick, 2008), Fc^*^=kFc[1+0.001xFc^2^λ^3^/sin(2θ)]^-1/4^
Primary atom site location: structure-invariant direct methods	Extinction coefficient: 0.082

### Fractional atomic coordinates and isotropic or equivalent isotropic displacement parameters (Å^2^)

**Table:**

	*x*	*y*	*z*	*U*_iso_*/*U*_eq_	
Pb1	0.12243 (2)	0.097018 (5)	0.352944 (18)	0.03251 (6)	
Pb2	0.63079 (2)	0.136517 (5)	0.498730 (18)	0.03285 (6)	
O1	0.0014 (5)	0.06035 (8)	0.5043 (3)	0.0425 (9)	
O2	0.2904 (5)	0.04888 (8)	0.4562 (3)	0.0422 (9)	
O3	−0.0208 (5)	0.12954 (8)	0.5322 (3)	0.0417 (9)	
O4	0.2740 (4)	0.11793 (8)	0.5341 (3)	0.0382 (8)	
O5	0.4697 (5)	0.10648 (9)	0.2967 (3)	0.0431 (9)	
O6	0.7648 (4)	0.11863 (8)	0.3157 (3)	0.0361 (8)	
O7	0.7820 (5)	0.18516 (8)	0.4032 (4)	0.0456 (9)	
O8	0.4875 (5)	0.17631 (9)	0.3567 (4)	0.0532 (11)	
O9	0.1259 (5)	0.15838 (9)	0.2844 (4)	0.0536 (11)	
H9A	0.2336	0.1648	0.2856	0.064*	
H9B	0.0480	0.1635	0.3359	0.064*	
O10	0.6329 (5)	0.07555 (8)	0.5507 (3)	0.0435 (9)	
H10B	0.5587	0.0665	0.5043	0.052*	
H10A	0.7351	0.0704	0.5243	0.052*	
C1	0.1464 (7)	0.04397 (11)	0.5191 (5)	0.0350 (12)	
C2	0.1449 (7)	0.01859 (11)	0.6140 (5)	0.0327 (11)	
C3	−0.0085 (7)	0.01413 (12)	0.6892 (5)	0.0419 (13)	
H3A	−0.1126	0.0268	0.6775	0.050*	
C4	−0.0118 (8)	−0.00864 (13)	0.7814 (5)	0.0449 (14)	
C5	0.1437 (9)	−0.02741 (13)	0.7950 (6)	0.0501 (15)	
H5A	0.1450	−0.0428	0.8563	0.060*	
C6	0.2948 (8)	−0.02395 (13)	0.7213 (6)	0.0483 (15)	
H6A	0.3966	−0.0372	0.7319	0.058*	
C7	0.2988 (8)	−0.00088 (12)	0.6303 (5)	0.0416 (13)	
H7A	0.4032	0.0016	0.5807	0.050*	
C8	−0.1813 (10)	−0.01275 (18)	0.8623 (7)	0.080 (2)	
H8A	−0.1591	−0.0294	0.9210	0.121*	
H8B	−0.2874	−0.0178	0.8107	0.121*	
H8C	−0.2050	0.0063	0.9066	0.121*	
C9	0.1354 (7)	0.13040 (11)	0.5855 (4)	0.0297 (11)	
C10	0.1578 (7)	0.14561 (12)	0.7091 (5)	0.0367 (12)	
C11	0.0214 (9)	0.16600 (13)	0.7525 (6)	0.0514 (15)	
H11A	−0.0821	0.1705	0.7024	0.062*	
C12	0.0388 (12)	0.17949 (16)	0.8690 (7)	0.072 (2)	
C13	0.1933 (15)	0.1719 (2)	0.9412 (7)	0.093 (3)	
H13A	0.2058	0.1805	1.0205	0.111*	
C14	0.3266 (12)	0.1524 (2)	0.8998 (7)	0.081 (2)	
H14A	0.4297	0.1481	0.9505	0.097*	
C15	0.3122 (9)	0.13865 (15)	0.7832 (5)	0.0563 (17)	
H15A	0.4040	0.1251	0.7551	0.068*	
C16	−0.1079 (14)	0.2018 (2)	0.9145 (9)	0.131 (4)	
H16A	−0.0742	0.2091	0.9965	0.197*	
H16B	−0.2258	0.1912	0.9175	0.197*	
H16C	−0.1171	0.2193	0.8586	0.197*	
C17	0.6265 (6)	0.10896 (11)	0.2509 (5)	0.0296 (11)	
C18	0.6596 (7)	0.10073 (11)	0.1176 (5)	0.0308 (11)	
C19	0.5301 (8)	0.08355 (12)	0.0511 (5)	0.0425 (13)	
H19A	0.4204	0.0776	0.0896	0.051*	
C20	0.5586 (10)	0.07500 (14)	−0.0711 (6)	0.0558 (17)	
C21	0.7238 (11)	0.08378 (15)	−0.1256 (6)	0.0634 (19)	
H21A	0.7476	0.0777	−0.2072	0.076*	
C22	0.8544 (10)	0.10140 (15)	−0.0611 (6)	0.0612 (18)	
H22A	0.9636	0.1076	−0.0999	0.073*	
C23	0.8229 (8)	0.10979 (13)	0.0606 (5)	0.0420 (13)	
H23A	0.9110	0.1215	0.1045	0.050*	
C24	0.4110 (11)	0.05680 (17)	−0.1440 (7)	0.090 (3)	
H24A	0.4543	0.0528	−0.2269	0.135*	
H24B	0.2980	0.0688	−0.1483	0.135*	
H24C	0.3875	0.0373	−0.1027	0.135*	
C25	0.6358 (8)	0.19171 (13)	0.3421 (5)	0.0413 (13)	
C26	0.6358 (8)	0.21833 (13)	0.2526 (5)	0.0437 (14)	
C27	0.7935 (9)	0.23678 (13)	0.2416 (5)	0.0500 (15)	
H27A	0.8980	0.2324	0.2908	0.060*	
C28	0.7986 (11)	0.26149 (15)	0.1590 (6)	0.0643 (19)	
C29	0.6435 (15)	0.26682 (19)	0.0853 (7)	0.091 (3)	
H29A	0.6446	0.2832	0.0283	0.109*	
C30	0.4891 (14)	0.2487 (2)	0.0936 (8)	0.095 (3)	
H30A	0.3868	0.2526	0.0417	0.114*	
C31	0.4833 (10)	0.22464 (17)	0.1785 (7)	0.069 (2)	
H31A	0.3758	0.2126	0.1857	0.083*	
C32	0.9668 (12)	0.28192 (18)	0.1483 (8)	0.107 (3)	
H32A	0.9438	0.2978	0.0865	0.161*	
H32B	1.0717	0.2695	0.1242	0.161*	
H32C	0.9928	0.2916	0.2274	0.161*	

### Atomic displacement parameters (Å^2^)

**Table:**

	*U*^11^	*U*^22^	*U*^33^	*U*^12^	*U*^13^	*U*^23^
Pb1	0.02451 (10)	0.04213 (12)	0.03088 (11)	−0.00082 (8)	0.00003 (7)	−0.00184 (9)
Pb2	0.02385 (9)	0.04156 (12)	0.03319 (11)	0.00000 (8)	0.00229 (8)	−0.00581 (9)
O1	0.034 (2)	0.042 (2)	0.052 (2)	0.0036 (17)	0.0058 (18)	0.0068 (18)
O2	0.0285 (19)	0.049 (2)	0.050 (2)	−0.0036 (16)	0.0087 (17)	0.0043 (18)
O3	0.034 (2)	0.053 (2)	0.038 (2)	0.0047 (17)	−0.0067 (17)	−0.0090 (18)
O4	0.0249 (18)	0.049 (2)	0.041 (2)	−0.0005 (16)	0.0056 (16)	−0.0044 (18)
O5	0.0280 (19)	0.059 (2)	0.042 (2)	−0.0028 (17)	0.0080 (17)	−0.0055 (19)
O6	0.0273 (18)	0.050 (2)	0.031 (2)	−0.0020 (16)	−0.0003 (15)	−0.0068 (17)
O7	0.037 (2)	0.042 (2)	0.058 (3)	−0.0016 (17)	−0.0059 (19)	0.0046 (19)
O8	0.039 (2)	0.052 (2)	0.068 (3)	−0.0060 (19)	−0.012 (2)	0.005 (2)
O9	0.035 (2)	0.069 (3)	0.057 (3)	−0.007 (2)	0.0047 (19)	0.001 (2)
O10	0.033 (2)	0.053 (2)	0.045 (2)	0.0014 (17)	0.0021 (17)	−0.0025 (18)
C1	0.032 (3)	0.034 (3)	0.039 (3)	−0.005 (2)	0.000 (2)	−0.004 (2)
C2	0.036 (3)	0.030 (3)	0.033 (3)	−0.005 (2)	−0.001 (2)	−0.004 (2)
C3	0.034 (3)	0.041 (3)	0.051 (4)	−0.003 (2)	0.002 (3)	−0.006 (3)
C4	0.047 (3)	0.044 (3)	0.044 (4)	−0.010 (3)	0.003 (3)	0.001 (3)
C5	0.069 (4)	0.034 (3)	0.047 (4)	−0.003 (3)	−0.007 (3)	0.005 (3)
C6	0.052 (4)	0.038 (3)	0.055 (4)	0.008 (3)	−0.002 (3)	0.005 (3)
C7	0.041 (3)	0.037 (3)	0.047 (4)	0.008 (2)	0.004 (3)	−0.005 (3)
C8	0.069 (5)	0.098 (6)	0.074 (5)	−0.007 (4)	0.018 (4)	0.030 (4)
C9	0.028 (3)	0.035 (3)	0.026 (3)	−0.002 (2)	−0.002 (2)	0.002 (2)
C10	0.041 (3)	0.039 (3)	0.031 (3)	−0.007 (2)	−0.004 (2)	0.004 (2)
C11	0.064 (4)	0.046 (3)	0.044 (4)	0.005 (3)	0.003 (3)	−0.008 (3)
C12	0.111 (6)	0.056 (4)	0.049 (4)	−0.004 (4)	0.008 (4)	−0.013 (4)
C13	0.151 (9)	0.081 (6)	0.045 (5)	−0.023 (6)	−0.015 (5)	−0.022 (4)
C14	0.105 (7)	0.093 (6)	0.044 (4)	−0.022 (5)	−0.029 (4)	0.003 (4)
C15	0.058 (4)	0.073 (4)	0.038 (4)	−0.003 (3)	−0.016 (3)	0.004 (3)
C16	0.178 (10)	0.098 (7)	0.119 (8)	0.041 (7)	0.035 (7)	−0.058 (6)
C17	0.025 (2)	0.034 (3)	0.030 (3)	0.002 (2)	0.002 (2)	0.001 (2)
C18	0.031 (3)	0.027 (3)	0.034 (3)	0.004 (2)	0.005 (2)	0.002 (2)
C19	0.056 (4)	0.041 (3)	0.030 (3)	−0.006 (3)	−0.001 (3)	0.001 (2)
C20	0.086 (5)	0.044 (4)	0.037 (4)	−0.009 (3)	−0.008 (3)	−0.008 (3)
C21	0.106 (6)	0.052 (4)	0.033 (4)	−0.005 (4)	0.020 (4)	−0.005 (3)
C22	0.073 (5)	0.066 (4)	0.046 (4)	−0.005 (4)	0.026 (3)	0.005 (3)
C23	0.043 (3)	0.045 (3)	0.038 (3)	−0.002 (3)	0.003 (3)	0.004 (3)
C24	0.139 (8)	0.079 (5)	0.052 (5)	−0.039 (5)	−0.021 (5)	−0.021 (4)
C25	0.039 (3)	0.040 (3)	0.046 (4)	0.000 (3)	−0.002 (3)	−0.005 (3)
C26	0.053 (4)	0.038 (3)	0.040 (3)	0.007 (3)	−0.004 (3)	−0.007 (3)
C27	0.065 (4)	0.043 (3)	0.042 (4)	0.007 (3)	0.005 (3)	0.000 (3)
C28	0.101 (6)	0.045 (4)	0.047 (4)	0.013 (4)	0.018 (4)	0.002 (3)
C29	0.148 (9)	0.069 (5)	0.057 (5)	0.025 (6)	0.020 (6)	0.025 (4)
C30	0.107 (7)	0.104 (7)	0.074 (6)	0.021 (6)	−0.022 (5)	0.034 (5)
C31	0.064 (5)	0.077 (5)	0.065 (5)	0.000 (4)	−0.014 (4)	0.011 (4)
C32	0.132 (8)	0.070 (5)	0.120 (8)	−0.018 (5)	0.045 (6)	0.028 (5)

### Geometric parameters (Å, °)

**Table:**

Pb1—O4	2.386 (3)	C10—C15	1.387 (7)
Pb1—O1	2.424 (3)	C10—C11	1.395 (7)
Pb1—O3	2.594 (3)	C11—C12	1.380 (8)
Pb1—O5	2.603 (3)	C11—H11A	0.9300
Pb1—O2	2.622 (4)	C12—C13	1.381 (11)
Pb1—O9	2.724 (4)	C12—C16	1.505 (10)
Pb1—O6^i^	2.751 (3)	C13—C14	1.348 (11)
Pb2—O6	2.325 (3)	C13—H13A	0.9300
Pb2—O8	2.494 (4)	C14—C15	1.384 (9)
Pb2—O3^ii^	2.538 (3)	C14—H14A	0.9300
Pb2—O7	2.565 (4)	C15—H15A	0.9300
Pb2—O10	2.665 (3)	C16—H16A	0.9600
Pb2—O4	2.712 (3)	C16—H16B	0.9600
Pb2—O5	2.757 (4)	C16—H16C	0.9600
O1—C1	1.263 (6)	C17—C18	1.493 (7)
O2—C1	1.258 (6)	C18—C19	1.376 (7)
O3—C9	1.251 (5)	C18—C23	1.384 (7)
O3—Pb2^i^	2.538 (3)	C19—C20	1.377 (8)
O4—C9	1.261 (5)	C19—H19A	0.9300
O5—C17	1.238 (5)	C20—C21	1.380 (9)
O6—C17	1.273 (5)	C20—C24	1.521 (8)
O6—Pb1^ii^	2.751 (3)	C21—C22	1.381 (9)
O7—C25	1.261 (6)	C21—H21A	0.9300
O8—C25	1.263 (6)	C22—C23	1.373 (8)
O9—H9A	0.8200	C22—H22A	0.9300
O9—H9B	0.8200	C23—H23A	0.9300
O10—H10B	0.8200	C24—H24A	0.9600
O10—H10A	0.8200	C24—H24B	0.9600
C1—C2	1.487 (7)	C24—H24C	0.9600
C2—C3	1.386 (7)	C25—C26	1.488 (8)
C2—C7	1.392 (7)	C26—C31	1.370 (8)
C3—C4	1.387 (7)	C26—C27	1.386 (8)
C3—H3A	0.9300	C27—C28	1.378 (8)
C4—C5	1.380 (8)	C27—H27A	0.9300
C4—C8	1.513 (8)	C28—C29	1.375 (11)
C5—C6	1.358 (8)	C28—C32	1.495 (10)
C5—H5A	0.9300	C29—C30	1.355 (11)
C6—C7	1.388 (7)	C29—H29A	0.9300
C6—H6A	0.9300	C30—C31	1.375 (10)
C7—H7A	0.9300	C30—H30A	0.9300
C8—H8A	0.9600	C31—H31A	0.9300
C8—H8B	0.9600	C32—H32A	0.9600
C8—H8C	0.9600	C32—H32B	0.9600
C9—C10	1.482 (7)	C32—H32C	0.9600
			
O4—Pb1—O1	82.05 (12)	C4—C8—H8B	109.5
O4—Pb1—O3	51.51 (11)	H8A—C8—H8B	109.5
O1—Pb1—O3	72.68 (12)	C4—C8—H8C	109.5
O4—Pb1—O5	72.72 (11)	H8A—C8—H8C	109.5
O1—Pb1—O5	127.44 (12)	H8B—C8—H8C	109.5
O3—Pb1—O5	118.56 (11)	O3—C9—O4	119.7 (5)
O4—Pb1—O2	75.46 (12)	O3—C9—C10	120.4 (4)
O1—Pb1—O2	51.48 (11)	O4—C9—C10	119.9 (4)
O3—Pb1—O2	107.02 (11)	C15—C10—C11	120.2 (5)
O5—Pb1—O2	77.50 (11)	C15—C10—C9	119.5 (5)
O4—Pb1—O9	81.54 (12)	C11—C10—C9	120.2 (5)
O1—Pb1—O9	143.85 (12)	C12—C11—C10	120.5 (6)
O3—Pb1—O9	71.86 (12)	C12—C11—H11A	119.8
O5—Pb1—O9	77.05 (11)	C10—C11—H11A	119.8
O2—Pb1—O9	149.74 (11)	C11—C12—C13	118.1 (7)
O4—Pb1—O6^i^	113.97 (10)	C11—C12—C16	120.2 (8)
O1—Pb1—O6^i^	88.41 (11)	C13—C12—C16	121.8 (7)
O3—Pb1—O6^i^	63.23 (10)	C14—C13—C12	121.9 (7)
O5—Pb1—O6^i^	143.91 (11)	C14—C13—H13A	119.0
O2—Pb1—O6^i^	138.35 (10)	C12—C13—H13A	119.0
O9—Pb1—O6^i^	69.48 (10)	C13—C14—C15	121.0 (7)
O6—Pb2—O8	83.21 (13)	C13—C14—H14A	119.5
O6—Pb2—O3^ii^	70.29 (11)	C15—C14—H14A	119.5
O8—Pb2—O3^ii^	124.28 (12)	C14—C15—C10	118.4 (7)
O6—Pb2—O7	75.42 (12)	C14—C15—H15A	120.8
O8—Pb2—O7	51.46 (12)	C10—C15—H15A	120.8
O3^ii^—Pb2—O7	74.37 (12)	C12—C16—H16A	109.5
O6—Pb2—O10	81.57 (11)	C12—C16—H16B	109.5
O8—Pb2—O10	142.70 (12)	H16A—C16—H16B	109.5
O3^ii^—Pb2—O10	81.54 (11)	C12—C16—H16C	109.5
O7—Pb2—O10	151.05 (11)	H16A—C16—H16C	109.5
O6—Pb2—O4	115.06 (11)	H16B—C16—H16C	109.5
O8—Pb2—O4	84.44 (12)	O5—C17—O6	121.2 (5)
O3^ii^—Pb2—O4	151.11 (11)	O5—C17—C18	121.1 (4)
O7—Pb2—O4	134.31 (11)	O6—C17—C18	117.7 (4)
O10—Pb2—O4	71.75 (10)	C19—C18—C23	119.4 (5)
O6—Pb2—O5	50.10 (10)	C19—C18—C17	120.4 (5)
O8—Pb2—O5	70.96 (12)	C23—C18—C17	120.2 (5)
O3^ii^—Pb2—O5	117.25 (10)	C18—C19—C20	121.8 (6)
O7—Pb2—O5	103.86 (11)	C18—C19—H19A	119.1
O10—Pb2—O5	73.13 (11)	C20—C19—H19A	119.1
O4—Pb2—O5	65.60 (10)	C19—C20—C21	118.0 (6)
C1—O1—Pb1	98.1 (3)	C19—C20—C24	120.8 (6)
C1—O2—Pb1	88.9 (3)	C21—C20—C24	121.2 (6)
C9—O3—Pb2^i^	159.3 (3)	C20—C21—C22	121.2 (6)
C9—O3—Pb1	89.5 (3)	C20—C21—H21A	119.4
Pb2^i^—O3—Pb1	110.94 (12)	C22—C21—H21A	119.4
C9—O4—Pb1	99.2 (3)	C23—C22—C21	119.9 (6)
C9—O4—Pb2	133.5 (3)	C23—C22—H22A	120.1
Pb1—O4—Pb2	114.63 (13)	C21—C22—H22A	120.1
C17—O5—Pb1	169.4 (3)	C22—C23—C18	119.8 (6)
C17—O5—Pb2	84.1 (3)	C22—C23—H23A	120.1
Pb1—O5—Pb2	106.44 (12)	C18—C23—H23A	120.1
C17—O6—Pb2	103.8 (3)	C20—C24—H24A	109.5
C17—O6—Pb1^ii^	133.6 (3)	C20—C24—H24B	109.5
Pb2—O6—Pb1^ii^	112.57 (13)	H24A—C24—H24B	109.5
C25—O7—Pb2	91.9 (3)	C20—C24—H24C	109.5
C25—O8—Pb2	95.2 (3)	H24A—C24—H24C	109.5
Pb1—O9—H9A	109.2	H24B—C24—H24C	109.5
Pb1—O9—H9B	93.9	O7—C25—O8	121.1 (5)
H9A—O9—H9B	123.4	O7—C25—C26	119.8 (5)
Pb2—O10—H10B	109.3	O8—C25—C26	119.1 (5)
Pb2—O10—H10A	101.0	C31—C26—C27	119.0 (6)
H10B—O10—H10A	103.9	C31—C26—C25	121.1 (6)
O2—C1—O1	121.4 (5)	C27—C26—C25	119.8 (5)
O2—C1—C2	120.1 (5)	C28—C27—C26	121.3 (6)
O1—C1—C2	118.5 (5)	C28—C27—H27A	119.4
C3—C2—C7	118.7 (5)	C26—C27—H27A	119.4
C3—C2—C1	120.7 (5)	C29—C28—C27	117.9 (7)
C7—C2—C1	120.6 (5)	C29—C28—C32	120.4 (7)
C2—C3—C4	122.1 (5)	C27—C28—C32	121.7 (7)
C2—C3—H3A	118.9	C30—C29—C28	121.5 (7)
C4—C3—H3A	118.9	C30—C29—H29A	119.2
C5—C4—C3	117.5 (5)	C28—C29—H29A	119.2
C5—C4—C8	121.8 (6)	C29—C30—C31	120.2 (8)
C3—C4—C8	120.7 (5)	C29—C30—H30A	119.9
C6—C5—C4	121.7 (6)	C31—C30—H30A	119.9
C6—C5—H5A	119.1	C26—C31—C30	120.0 (7)
C4—C5—H5A	119.1	C26—C31—H31A	120.0
C5—C6—C7	120.7 (5)	C30—C31—H31A	120.0
C5—C6—H6A	119.6	C28—C32—H32A	109.5
C7—C6—H6A	119.6	C28—C32—H32B	109.5
C6—C7—C2	119.3 (5)	H32A—C32—H32B	109.5
C6—C7—H7A	120.4	C28—C32—H32C	109.5
C2—C7—H7A	120.4	H32A—C32—H32C	109.5
C4—C8—H8A	109.5	H32B—C32—H32C	109.5

Symmetry codes: (i) *x*−1, *y*, *z*; (ii) *x*+1, *y*, *z*.

### Hydrogen-bond geometry (Å, °)

**Table:**

*D*—H···*A*	*D*—H	H···*A*	*D*···*A*	*D*—H···*A*
O9—H9A···O8	0.82	2.03	2.805 (5)	158
O9—H9B···O7^i^	0.82	2.25	3.017 (5)	156
O10—H10B···O2	0.82	2.12	2.881 (5)	153
O10—H10A···O1^ii^	0.82	1.97	2.774 (5)	166

Symmetry codes: (i) *x*−1, *y*, *z*; (ii) *x*+1, *y*, *z*.
